# Micronutrient gaps during the complementary feeding period in seven countries in Southeast Asia: A Comprehensive Nutrient Gap Assessment

**DOI:** 10.1111/mcn.13577

**Published:** 2023-12-13

**Authors:** Jessica M. White, Elizabeth Drummond, Vasundhara Bijalwan, Anusara Singhkumarwong, Arvind Betigeri, Jessica Blankenship

**Affiliations:** ^1^ UNICEF East Asia and the Pacific Regional Office Bangkok Thailand; ^2^ World Food Programme Asia and the Pacific Regional Office Bangkok Thailand

**Keywords:** child nutrition, complementary feeding, dietary assessment tools, food and nutrient intake, micronutrients, Southeast Asia

## Abstract

The complementary feeding period is a critical stage of child development when micronutrient needs are high and challenging to meet. Understanding if specific micronutrient gaps exist during this period is critical for effective programming. A Comprehensive Nutrient Gap Assessment (CONGA) was conducted in seven countries in Southeast Asia to estimate gaps in micronutrients commonly lacking in the diets of children aged 6–23 months and to establish the certainty of available evidence for each identified gap. Sixty‐eight evidence sources were identified during this analysis, and 310 micronutrient‐specific data points were identified across all seven countries. Data points varied in recency, representativeness and evidence type. The CONGA methodology enabled the estimation of a gap burden rating for each micronutrient in each country, as well as a rating of their evidence certainty. Micronutrient gaps were identified in vitamin D, zinc and iron and a potential gap was identified in calcium during the complementary feeding period in the region. Evidence relevant to intake and deficiency of folate, vitamin B_12_, thiamine, niacin, vitamin C and vitamin B_6_ was limited across the region. Proven strategies to address these gaps include increasing the availability and consumption of nutrient‐dense foods, micronutrient supplementation, large‐scale fortification of staple foods and condiments and point‐of‐use fortification through multiple micronutrient powders and fortified speciality foods. More recent data on micronutrient availability, intake and deficiency is urgently needed in Southeast Asia.

## Key messages

- Older infants and young children aged 6–23 months experience micronutrient gaps during the complementary feeding period in Southeast Asia. Synthesis of evidence compiled across seven countries in the region identified gaps in vitamin D, zinc and iron and a potential gap in calcium during this critical period.
- National nutrition policies and programmes should consider multiple strategies to address these micronutrient gaps.
- Over half of the data points relevant to nutrient deficiency and intake compiled from the seven countries are over a decade old. More recent data on micronutrient availability, intake and deficiency is urgently needed in Southeast Asia.

## 1 INTRODUCTION

Insufficient quality, quantity and diversity of foods during the complementary feeding period—between 6 months and 2 years of age—can negatively impact children's physical and cognitive development (Dewey, 2001; Dewey, 2013; Lutter et al., 2021). Yet, in Southeast Asia (SEA), only half of the children aged 6–23 months consume the minimum recommendation for a diverse diet, and only 72% are fed the minimum recommended number of times per day (United Nations Children's Fund, Division of Data, Analysis, Planning and Monitoring, 2022). Suboptimal diets in early life have contributed to a triple burden of malnutrition—a concurrence of undernutrition, overweight and micronutrient deficiencies—in the region (Blankenship et al., 2020). An estimated 27% of children under 5 years of age are stunted, 8.2% are wasted and 7.5% are overweight in SEA (UNICEF, WHO, & The World Bank, 2021), and an estimated 58% of children aged 6–59 months in East Asia and the Pacific—approximately 85 million children—are deficient in at least one essential micronutrient (zinc, iron or vitamin A) (Stevens et al., 2022).

Improvements in the diets of children aged 6–23 months can help reduce the burden of micronutrient deficiencies in the region and accelerate progress in the reduction of other forms of malnutrition. However, robust evidence indicative of nutrient deficiencies or nutrient intake in young children is often unavailable, underused or misinterpreted (Beal, White, Arsenault, Okronipa, Hinnouho, Murira, Torlesse et al., 2021; White et al., 2021). As a result, a government's ability to design effective policies and programmes to improve micronutrient intake—such as legislation on mandatory fortification—may be limited. The existing evidence base may also exclude data relevant to all micronutrients commonly lacking during the complementary feeding period (PAHO & WHO, 2003) as data on intake and deficiency is often limited to a minimal selection of micronutrients (Beal, White, Arsenault, Okronipa, Hinnouho, Murira, Torlesse et al., 2021; Stevens et al., 2022; White et al., 2021).

The Comprehensive Nutrient Gap Assessment (CONGA) is a methodology designed to identify nutrient gaps (i.e., shortfalls in the diet that may lead to deficiency or adverse health outcomes) in a defined population and to estimate the public health significance of these gaps and the certainty of evidence they are based on. To do this, CONGA guides the compilation and synthesis of a variety of evidence types relevant to nutrient gaps (e.g., biochemical markers, individual and household nutrient intake) from disparate data sources that may vary in representativeness, content and recency. Compiling and synthesising a wide range of evidence types and sources can provide insight into the depth and breadth of the relevant evidence base and provide a more comprehensive understanding of nutrient gaps than a review of only one evidence type or of a subset of the evidence base only (Beal, White, Arsenault, Okronipa, Hinnouho, & Morris, 2021; Beal, White, Arsenault, Okronipa, Hinnouho, Murira, Torlesse et al., 2021).

To better understand micronutrient gaps among children aged 6–23 months in SEA, CONGA was implemented in seven countries in the region: Cambodia, Indonesia, Lao People's Democratic Republic (PDR), Malaysia, the Philippines, Thailand and Viet Nam. The objectives of this analysis were to (1) estimate micronutrient gaps during the complementary feeding period and (2) establish the certainty of available evidence for each gap in each country.

## 2 METHODS

The CONGA methodology is described fully in a separate publication (Beal, White, Arsenault, Okronipa, Hinnouho, & Morris, 2021) and is summarised here. CONGA was used to estimate gaps in micronutrients commonly lacking in the diets of children aged 6–23 months: iron, vitamin A, zinc, calcium, iodine, vitamin B_1_ (thiamine), niacin, vitamin B_12_, vitamin B_6_, folate, vitamin C and vitamin D (PAHO & WHO, 2003). With the exception of vitamin D, these same micronutrients were included in two previous CONGA analyses focused on the complementary feeding period in Eastern and Southern Africa and South Asia (Beal, White, Arsenault, Okronipa, Hinnouho, Murira, Torlesse et al., 2021; White et al., 2021). Vitamin D was added for this analysis as there is a growing body of evidence that deficiency is common in SEA (Oktaria et al., 2022).

### 2.1 Literature search and data extraction

The CONGA methodology prioritises five evidence types to estimate nutrient gaps: (1) biological, clinical and functional markers, (2) nutrient adequacy of individual diets, (3) nutrient adequacy of household diets, (4) nutrient adequacy of national food supplies and (5) nutrient‐informative food group intake of individuals or households (Beal, White, Arsenault, Okronipa, Hinnouho, & Morris, 2021). A literature search was conducted to identify relevant evidence sources for these five evidence types in all seven countries. Evidence was identified by searching relevant global databases (e.g., UNICEF Global Databases, the Iodine Global Network Global Scorecard) and by using keyword searches in PubMed, Google Scholar and Google. Keyword search terms covered the area of interest (e.g., micronutrient deficiencies in children aged 6–23 months) and relevant survey types or grey literature (e.g., national nutrition surveys; Demographic and Health Surveys [DHS]).

Evidence specific to children aged 6–23 months was prioritised. However, evidence from other age groups that (1) overlapped with this age group (e.g., children aged 6–59 months), (2) were close to but did not overlap (e.g., children aged 5–9 years) or (3) were not close to but were relevant to child nutrition (e.g., women of reproductive age [WRA]) was considered. Evidence that did not fall into the five priority evidence types but was still relevant to the diets of children aged 6–23 months (e.g., linear programming to identify problem nutrients) was still compiled and classified as ‘other’ evidence. Evidence representative only of highly vulnerable population groups (e.g., children in a refugee camp) was not considered. UNICEF country offices and government colleagues in the seven countries were consulted to determine whether evidence sources were missed.

Data points from identified evidence sources were extracted and captured in country‐specific spreadsheets. Where there were multiple data points for a specific evidence type from similar evidence sources (e.g., zinc deficiency prevalence from repeated national nutrition surveys), the most recent data point was used and the older data was recorded in a ‘comment’ box to provide additional context (e.g., temporal trends). Where there were multiple data points for a specific evidence type from the same source but for different age groups (e.g., for both children 6–59 months and WRA), the data point closest to children 6–23 months was selected and the others were captured in the ‘comment’ box. If there were multiple data points for a specific evidence type from different sources (e.g., zinc deficiency prevalence from both a DHS and a national nutrition survey), each data point was captured separately.

### 2.2 Assignment of implied nutrient burden (INB) ratings

Per CONGA methodology, an ‘INB’ rating (i.e., public health significance) of negligible, low, moderate or high was assigned to each data point using prevalence and mean ranges for commonly available population‐level indicators for the five priority evidence types (e.g., a data point for vitamin A deficiency prevalence >20% was assigned an INB rating of ‘high’). Each rating had an associated weight score: negligible (0), low (1), moderate (2) and high (3). The CONGA methodology does not provide suggested INB ratings for ‘other’ evidence types or for biological, clinical and functional markers of deficiency for calcium, thiamine or vitamin D. Therefore, ‘other’ evidence data points and prevalence estimates for calcium or thiamine deficiency were assigned an INB rating of ‘not applicable’ (N/A) and no weight score was assigned. For vitamin D deficiency, global guidance on population thresholds and biochemical markers was used to determine prevalence ranges for INB ratings. Detail on INB ratings for each evidence type and micronutrient is provided in Supporting Information: Table S1.

### 2.3 Metadata

To document and account for variations in evidence type, recency, relevance and representativeness of each data point, five types of metadata were extracted, recorded and assigned a weight in the country‐specific spreadsheets: (1) evidence type, (2) geographic representation, (3) year of data collection, (4) age and sex group and (5) sample size (Table 1) (Beal, White, Arsenault, Okronipa, Hinnouho, & Morris, 2021). The ‘year of data collection’ category was adjusted for this analysis to allow for consideration of data points collected up to 12 years ago (previous CONGA used a cut‐off of 10 years) (Beal, White, Arsenault, Okronipa, Hinnouho, Murira, Torlesse et al., 2021; White et al., 2021). The literature search was completed in 2022; therefore, data collected from 2010 was eligible for consideration.

**Table 1:** Metadata and assigned weights.^a^

Metadata type	Metadata and assigned weight score
Evidence type	Biological, clinical and functional markers (5). Nutrient adequacy of individual diets (3). Nutrient adequacy of household diets (2). Nutrient adequacy of national food supplies (1). Nutrient‐informative food group intake of individuals or households (1). Other (N/A).
Geographic representation^b^	Representative of the entire geographic area of interest (5). Representative of 75%–99% of the total population in the geographic area of interest (4). Representative of 50%–74% of the total population in the geographic area of interest (3). Representative of 25%–49% of the total population in the geographic area of interest (2). Representative of 10%–24% of the total population in the geographic area of interest (1). Representative of <10% of the total population in the geographic area of interest (N/A).
Recency of data collection	<4 years (5). 4–5 years (4). 6–7 years (3). 8–9 years (2). 10–12 years (1). >12 years (N/A).
Age and sex representation	Estimates for exact age and sex group of interest (5). Estimates for either a subgroup within the age and sex group of interest representing at least half of the group or an age and sex group, at least half of which includes the age and sex group of interest (4). Estimates for an age and sex group that includes the age and sex group of interest, but less than half of which includes the age and sex group of interest; or estimates for an age and sex group that is similar to but excludes the age and sex group of interest entirely (3). Household or food balance sheet estimates, less than half of which includes the age and sex group of interest (2). Estimates for an age and sex group that is similar to but excludes the age and sex group of interest entirely (1). Estimates for an age and sex group that excludes and is not near to the age and sex group of interest entirely (N/A).
Sample size	>1000 (5). 500–1000 (4). 300–499 (3). 100–299 (2). 50–99 (1). Based on national food supplies (0). <50 (N/A)

^a^ Numbers in parentheses represent the weight score.

^b^ Divide the population total that the study is representative of by the total population of the geographic area of interest.

Metadata weight scores were used to generate overall weight scores for each data point by multiplying the evidence type metadata weight score by the combined weight scores of its other four metadata categories (with a maximum possible weight score of 100 and a minimum possible score of 4). Thus, the most robust data points (e.g., the most recent, representative and relevant) had greater weight scores and the less robust (e.g., older, subnational and small sample size) had lower weight scores. Data points were *not* assigned a weight score if they were (1) categorised as ‘other’ evidence, (2) representative of <10% of the national population, (3) collected before 2010, (4) representative of an age and sex group that excluded and was not near to children aged 6–23 months or (5) based on a sample size <50.

### 2.4 Nutrient gap burden rating

A nutrient gap burden rating (i.e., an estimate of the public health significance of a nutrient gap) was then determined for each of the 12 micronutrients assessed using both INB ratings and data point weight scores. During this step, CONGA requires at least two experts to jointly review all evidence. Four researchers with expertise in infant and young child nutrition and knowledge of the SEA context reviewed the data points and at least two researchers jointly reviewed each country.

Determination of the nutrient gap burden rating is a two‐step process. First, CONGA generated a weighted nutrient gap burden score for each of the 12 micronutrients. For each data point relevant to a particular micronutrient, the INB rating weight score was multiplied by the overall data point weight score (thus, data points without weight scores, such as for ‘other’ evidence, were omitted from this calculation). The sum of all these values was then divided by the sum of the data point weights, with possible nutrient gap burden scores ranging from 0.0 to 3.0. Each weighted nutrient gap burden score was then assigned a rating of public health significance based on its value: negligible (<0.5), low (0.5–1.49), moderate (1.50–2.49) or high (≥2.5) (Beal, White, Arsenault, Okronipa, Hinnouho, & Morris, 2021).

Next, researchers reviewed these weighted nutrient gap burden score ratings alongside the totality of evidence collated, including data points that did receive weight scores (e.g., ‘other’ evidence) and additional information in the comment box for each data point (e.g., indicator trends). Researchers qualitatively considered all evidence to determine whether the final nutrient gap burden rating should deviate from the weighted burden score rating. Final ratings of negligible, low, moderate or high were assigned to each of the 12 micronutrients in each country, and any deviation from the weighted burden score ratings was documented. A rating of ‘unknown’ was assigned if no data points were identified for the micronutrient during the literature search.

### 2.5 Evidence certainty rating

Two steps were used to determine the certainty of evidence for each final nutrient gap burden rating. First, a criteria‐based rating of certainty (low, moderate or high) was assigned. This rating only considers the data points that received weight scores and is based on the level of agreement between the INB ratings (e.g., variation or consistency in INB ratings for a micronutrient) and the evidence weight score for the data points (see Supporting Information: Table S2). Next, researchers conducted a qualitative review, considering all available evidence, to determine whether the final rating should deviate from the criteria‐based rating. Any deviations were documented. A rating of ‘unknown’ was assigned where there was no data available to review.

Per CONGA methodology, micronutrients with a final nutrient gap burden rating of moderate or high and an evidence certainty rating of moderate or high were labelled as ‘identified micronutrient gaps’ and considered as high‐priority. Micronutrients with a final nutrient gap burden rating of moderate or high and a certainty‐of‐evidence rating of low were considered to be ‘potential micronutrient gaps’ (Beal, White, Arsenault, Okronipa, Hinnouho, & Morris, 2021).

Supporting Information: Table S4 includes all seven country‐level spreadsheets with extracted data points, data sources, metadata, weight scores and final nutrient gap burden and certainty ratings.

## 3 RESULTS

A total of 68 unique evidence sources were identified during the literature search (see Supporting Information: Table S3) from which data points and metadata were extracted. A total of 310 data points were extracted across all seven countries. The vast majority (*n* = 236) of data points were nationally representative. More than half (*n* = 170) were collected between 2010 and 2012, 99 (31.9%) were collected between 2013 and 2018 and 29 (9.4%) were collected between 2019 and 2022. Most data points were either specifically representative of children aged 6–23 months (*n* = 133) or based on household or food balance sheet estimates (*n* = 104). However, only 134 of the 310 data points met the criteria to be assigned a weight score.

The availability of data points varied between countries, ranging from 54 in Cambodia to 29 in Malaysia (Table 2). Availability also varied by micronutrient, ranging from 52 for iron to 10 for vitamin D (Table 3). Data also varied by evidence type. For example, the number of biological, clinical or functional marker data points (hereafter referred to as deficiency prevalence data) varied between countries and nutrients. Sixty‐one deficiency prevalence data points were identified, and 31 met the criteria for a weight score. Cambodia produced the most deficiency prevalence data points (*n* = 15), including iron, vitamin A, zinc, calcium, vitamin B_12_, thiamine and vitamin D, with all deficiency prevalence data points representative of a subgroup of young children (see Supporting Information: Table S4).

**Table 2:** Number of data points identified for the CONGA, and that met criteria for a weight score, by country and evidence type.^a^

Countries	Evidence types						Total
	Biological, clinical and functional markers	Nutrient adequacy of individual diets	Nutrient adequacy of household diets	Nutrient adequacy of national food supplies	Nutrient‐informative food group intake of individuals or households	Other	
Cambodia	15 (7)	0 (0)	0 (0)	10 (10)	3 (3)	26 (0)	54 (20)
Indonesia	6 (4)	0 (0)	0 (0)	10 (10)	4 (3)	33 (0)	53 (17)
Philippines	8 (4)	10 (10)	2 (2)	10 (10)	3 (3)	18 (0)	51 (29)
Lao PDR	7 (1)	6 (6)	0 (0)	10 (10)	3 (2)	20 (0)	46 (19)
Viet Nam	10 (8)	0 (0)	0 (0)	10 (10)	2 (2)	18 (0)	40 (20)
Thailand	7 (4)	0 (0)	0 (0)	10 (10)	2 (2)	18 (0)	37 (16)
Malaysia	8 (3)	0 (0)	0 (0)	10 (10)	2 (0)	9 (0)	29 (13)
Total	61 (31)	16 (16)	2 (2)	70 (70)	19 (15)	142 (0)	310 (134)

Abbreviations: CONGA, Comprehensive Nutrient Gap Assessment; PDR, People's Democratic Republic.

^a^ The parentheses enclose the number of data points that qualified for a weight score.

**Table 3:** Number of data points identified for the CONGA, and that met criteria for a weight score, by micronutrient and evidence type.^a^

Micronutrients assessed	Evidence types						Total
	Biological, clinical and functional markers	Nutrient adequacy of individual diets	Nutrient adequacy of household diets	Nutrient adequacy of national food supplies	Nutrient‐informative food group intake of individuals or households	Other	
Iron	13 (7)	2 (2)	1 (1)	7 (7)	6 (5)	23 (0)	52 (22)
Vitamin A	14 (7)	2 (2)	0 (0)	7 (7)	4 (3)	19 (0)	46 (19)
Calcium	2 (0)	2 (2)	1 (1)	7 (7)	0 (0)	23 (0)	35 (10)
Zinc	5 (3)	1 (1)	0 (0)	7 (7)	0 (0)	20 (0)	33 (11)
Vitamin B_12_	2 (1)	1 (1)	0 (0)	7 (7)	0 (0)	12 (0)	22 (9)
Folate	4 (2)	1 (1)	0 (0)	7 (7)	0 (0)	10 (0)	22 (10)
Vitamin C	0 (0)	2 (2)	0 (0)	7 (7)	0 (0)	11 (0)	20 (9)
Iodine	10 (5)	0 (0)	0 (0)	0 (0)	9 (7)	0 (0)	19 (12)
Vitamin B_1_	2 (0)	2 (2)	0 (0)	7 (7)	0 (0)	8 (0)	19 (9)
Niacin	0 (0)	2 (2)	0 (0)	7 (7)	0 (0)	8 (0)	17 (9)
Vitamin B_6_	0 (0)	1 (1)	0 (0)	7 (7)	0 (0)	7 (0)	15 (8)
Vitamin D	9 (6)	0 (0)	0 (0)	0 (0)	0 (0)	1 (0)	10 (6)
Total	61 (31)	16 (16)	2 (2)	70 (70)	19 (15)	142 (0)	310 (134)

Abbreviation: CONGA, Comprehensive Nutrient Gap Assessment.

^a^ The parentheses enclose the number of data points that qualified for a weight score.

Across all countries, deficiency prevalence data points were most frequently available for vitamin A (*n* = 14), iron (*n* = 13), iodine (*n* = 10) and vitamin D (*n* = 9). Vitamin A and iron deficiency prevalence data were identified in all countries, with all but one (Lao PDR) having a nationally representative data point for each. All nationally representative iron deficiency data points were collected before 2015. Six countries had at least one nationally representative data point on iodine deficiency. Nine vitamin D deficiency data points were identified in six countries. Six of these were national and three were subnational. Except for Cambodia, all national vitamin D deficiency data were collected between 2010 and 2011. The representative age and population group for the micronutrient deficiency prevalence estimates varied. For example, the iron deficiency data point in the Philippines was representative only of children aged 6–9 years, and Cambodia and Thailand were the only countries to provide national iodine deficiency data points representative of children under 5 years of age (children aged 3–5 years in Thailand). Viet Nam, Cambodia and Indonesia had vitamin D deficiency data representative of an age range of children under 5 years. Apart from one data point, all vitamin D data identified for this analysis were deficiency prevalence estimates.

Five zinc deficiency data points were identified across four countries (Cambodia, Lao PDR, the Philippines and Viet Nam), of which three were nationally representative. Notably, in Viet Nam, the national zinc deficiency prevalence was 58% in children under 5, and in Cambodia, the national prevalence in children 6–72 months was 68%. Nationally representative calcium deficiency data points for subgroups of young children were identified in Cambodia and Viet Nam. Deficiency data for folate was identified in Cambodia, Lao PDR and Viet Nam and for thiamine and vitamin B_12_ in Cambodia and Lao PDR (Supporting Information: Table S4). However, the calcium and thiamine deficiency data points did not receive weight scores because there are no suggested INB ratings for these deficiencies in CONGA. No deficiency data was identified in any country for niacin, vitamin C or vitamin B_6_.

Evidence on nutrient adequacy of individual diets was limited, with six data points identified in Lao PDR and 11 data points identified in the Philippines. Nutrient‐informative food group estimates for individuals were available for vitamin A and iron (e.g., consumption of iron‐ and vitamin A‐rich foods) in most countries. Nutrient‐informative food group intake for households (e.g., household coverage of iodised salt) was available for all countries. Evidence on nutrient adequacy of household diets was only available in the Philippines, but for iron and calcium only.

Evidence on nutrient adequacy of national food supplies was available for all micronutrients except vitamin D and iodine in all seven countries. One evidence source provided all nutrient adequacy of national food supply data points for all micronutrients except calcium and zinc. This evidence source used food supply data from 2011 to estimate the percentage of the national population with inadequate access to a micronutrient based on its availability in the food supply (Beal et al., 2017). Food supply data was the sole source of information used for the weighted nutrient gap burden calculation for vitamin B_6_ in six countries. In total, 39 of the 84 weighted nutrient gap burden scores calculated in this analysis were based exclusively on national food supply data (primarily for niacin, thiamine, calcium and vitamins B_12_ and C, and to a lesser extent folate and zinc).

There were 142 ‘other’ evidence data points identified. These included estimates of intake of foods or food groups considered high in one or more micronutrients (e.g., dairy is high in calcium), linear programming results identifying limiting nutrients in the diets of young children (e.g., Optifood analyses), micronutrient supplementation coverage data and other evidence relevant to micronutrient intake or gaps. Data points on anaemia prevalence in young children were available in all countries but were classified as ‘other’ evidence rather than biological, clinical or functional markers, per CONGA methodology (Beal, White, Arsenault, Okronipa, Hinnouho, & Morris, 2021).

### 3.1 Ratings adjustments

When determining final ratings, researchers made seven adjustments to weighted nutrient gap burden score ratings and seven to criteria‐based certainty of evidence ratings. Two of the weighted nutrient gap burden score rating adjustments (iodine in Malaysia and vitamin D in the Philippines) were made because no data points qualified for a weight score and thus no weighted burden calculation or criteria‐based certainty ratings could be assigned. During the final review of evidence, researchers assigned a low rating for both the nutrient gap burden and evidence certainty. Three rating adjustments each were made for iron, vitamin A and vitamin D, two adjustments were made for iodine and one adjustment was made each for zinc, vitamin B_12_ and calcium. The justification for all rating adjustments is presented in Supporting Information: Table S4.

### 3.2 Final nutrient gap burden and evidence certainty ratings

**Figure 1:**
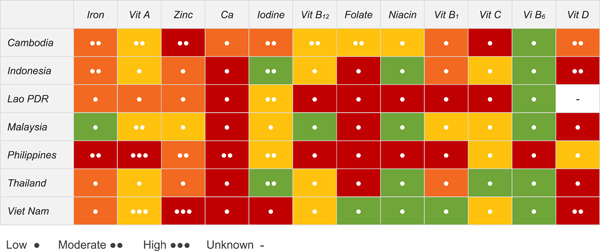
Final nutrient gap burden and certainty of evidence ratings for 12 micronutrients among children 6–23 months of age, by country. Nutrient gap burden is signified by colour: red (high burden), orange (moderate burden), yellow (low burden), green (negligible burden) or white (unknown burden). The number of circles (●) represents the certainty of evidence for the nutrient gap burden: three (high certainty), two (moderate certainty) or one (low certainty) evidence. And (‐) indicates certainty is unknown. Ca, calcium; PDR, People's Democratic Republic; Vit, vitamin.

The final nutrient gap burden and evidence certainty ratings are presented in Figure 1. Calcium received the highest nutrient burden gap ratings with an estimated high burden gap in six countries and a moderate burden gap in one. Gaps in vitamin D were also high, with estimated high burden gaps in four countries and moderate burden gaps in one. Gaps in zinc were estimated as high in two countries and moderate in four. Gaps in iron were high in two countries and moderate in an additional five. Gaps in vitamin A were high in one country and moderate in one (and low in five).

Most final nutrient gap burden ratings (62 out of 84) had a low certainty of evidence. The micronutrients with the highest certainty of evidence were vitamin A (two high evidence certainty ratings and two moderate) and zinc (one high evidence certainty rating and two moderate). Iodine had five moderate certainty of evidence ratings, and iron and vitamin D each had three moderate certainty ratings. All nutrient gap burden ratings for thiamine, niacin, vitamin B_6_ and vitamin C were based on low‐certainty evidence.

Identified micronutrient gaps (i.e., those with a final nutrient gap burden and certainty‐of‐evidence rating of at least moderate) and potential gaps (i.e., those with a final nutrient gap burden rating of at least moderate but a low certainty‐of‐evidence rating) varied between countries. Based on available evidence, vitamin D, zinc and iron were identified as micronutrient gaps across multiple countries, and calcium, iodine and vitamin A were identified as micronutrient gaps in one country each. Calcium was a potential micronutrient gap in six countries, receiving a high or moderate burden rating but with low certainty evidence. Vitamin A and iodine, however, were most frequently assigned a negligible or low final nutrient gap burden rating, often with a moderate to high certainty rating. Thus, while there may be gaps in individual countries, there does not appear to be a potential region‐wide gap for either micronutrient.

## 4 DISCUSSION

The complementary feeding period is a critical stage of development when micronutrient requirements are high and challenging to meet (Dewey, 2013). Preventing micronutrient deficiencies in children aged 6–23 months through relevant, context‐specific policies and programmes requires a comprehensive understanding of dietary gaps; however, relevant evidence and analyses to identify nutrient gaps are often lacking. The CONGA methodology applied to seven countries in SEA enabled the synthesis of multiple evidence types from disparate sources and provided a comprehensive picture of the magnitude and significance of micronutrient gaps in the diets of children aged 6–23 months and the depth, breadth and quality of the relevant evidence in the region.

Gaps in vitamin D, zinc and iron were identified across multiple countries in SEA and should be considered a priority for policy, programmes and research to improve micronutrient intake during the complementary feeding period. Vitamin D deficiency may cause rickets and delayed motor development in young children and osteomalacia in adulthood (Balasubramanian, 2011); zinc deficiency in early life is associated with impaired immune function and cognitive and motor development and increased risk of diarrhoea (Black, 1998; Semrad, 1999); and early iron deficiency can result in poorer cognitive, motor and social–emotional development (Lozoff, 2007; Stoltzfus, 2003). Calcium was identified as a potential micronutrient gap in SEA and should be prioritised during new data collection and evidence generation. Calcium deficiency can increase the risk of rickets; however, wider, long‐term health implications of calcium deficiency in young children are not well established (Pettifor, 2014; White et al., 2021).

The CONGA analyses in both Eastern and Southern Africa and South Asia also identified iron, zinc and calcium as micronutrient gaps among children aged 6–23 months (vitamin D was not assessed) (Beal, White, Arsenault, Okronipa, Hinnouho, Murira, Torlesse et al., 2021; White et al., 2021). These three micronutrients have been consistently observed as inadequate during the complementary feeding period across low‐ and middle‐income countries (Osendarp et al., 2016). Further, where deficiency prevalence data for iron and zinc is available, deficiency among children is common: An analysis of 24 nationally representative surveys from 22 countries across South Asia, SEA, Latin America, sub‐Saharan Africa and Europe found that iron and zinc deficiency among preschool‐aged children is frequently 20% or higher (Stevens et al., 2022).

A recent systematic review of vitamin D deficiency among children in SEA found that deficiency is common, particularly among newborns and female children residing in urban areas (Oktaria et al., 2022). Recent assessments have also identified high levels of vitamin D deficiency in children under 5 years of age in South Asia (Government of Pakistan, United Nations Children's Fund, 2019; Ministry of Health and Family Welfare, Government of India, UNICEF, & Population Council, 2019; Ministry of Public Health, United Nations Children's Fund, 2013) and in the wider population in Africa (Mogire et al., 2020). Preventative measures such as education on safe sun exposure, supplementation among children and pregnant women, or fortification may be required to improve vitamin D status in SEA and beyond.

The CONGA in SEA found that micronutrient gap burdens in iodine were frequently negligible or low, with moderate certainty evidence. For vitamin A, three countries were assigned a low burden with moderate‐to‐high evidence certainty. These findings suggest there may not be significant dietary gaps in these micronutrients during the complementary feeding period in the region. While the final nutrient gap burden ratings were mixed for folate, vitamin B_12_, thiamine, niacin, vitamin B_6_ and vitamin C, all, or nearly all, of these ratings were based on low certainty evidence. Despite recognition that these nutrients are commonly lacking during the complementary feeding period (PAHO & WHO, 2003), evidence of intake of and/or deficiency of these micronutrients was scarce in SEA, as also observed in Eastern and Southern Africa and South Asia (Beal, White, Arsenault, Okronipa, Hinnouho, Murira, Torlesse et al., 2021; White et al., 2021). It is therefore difficult to estimate the public health significance of these gaps with certainty.

Over half (59%) of all data points compiled for this analysis, and 46% of deficiency prevalence data points available, are over a decade old. A third of deficiency prevalence data points were from small subnational surveys, limiting their utility to determine national gaps. Sixty‐nine percent of the deficiency data points were for iron, vitamin A, zinc or iodine only. Only 18 data points on nutrient adequacy of individual or household diets were identified, highlighting a gap in the collection and use of dietary intake and consumption data. Across SEA, young children's diets are changing as the region experiences a nutrition transition (ASEAN, UNICEF, & WFP, 2022). Without more recent data relevant to nutrient gaps, it is difficult to monitor if and how micronutrient gaps and their risks are changing as children's diets shift.

Evidence‐based strategies are required to address the micronutrient gaps identified for children aged 6–23 months in this analysis. Relevant strategies include increasing the availability and consumption of nutrient‐dense foods, micronutrient supplementation, large‐scale fortification of staple foods and condiments and point‐of‐use fortification through multiple micronutrient powders (MNPs) and fortified speciality foods.

In SEA, healthy diets remain unaffordable for millions (FAO, IFAD, UNICEF, WFP, & WHO, 2020) and national food supplies are still dominated by starchy foods (ASEAN, UNICEF, & WFP, 2022). Increasing the availability and affordability of nutrient‐rich animal‐source foods and fruits and vegetables can help address micronutrient gaps. In SEA, foods rich in several of the identified micronutrient gaps in this analysis are primarily animal‐source foods (organs, crustaceans, goat, eggs, fresh and canned fish, beef, lamb, cow's milk, yoghurt and cheese) and dark leafy green vegetables (Ortenzi & Beal, 2021). No single food however is a high source for all identified micronutrient gaps. Therefore, the promotion of diverse diets, emphasising micronutrient‐dense foods, is essential to close micronutrient gaps during the complementary feeding period. Research is needed to understand context‐specific demand and supply‐side barriers to the consumption of micronutrient‐dense foods, including availability, affordability, accessibility or desirability (UNICEF, 2020). However, even when locally available foods are optimally used, the micronutrient needs of children aged 6–23 months may not be met (particularly for iron and zinc) and other strategies may be required to ensure adequate intake (Osendarp et al., 2016).

In addition to general diet improvements, other more targeted approaches are needed. Large‐scale supplementation can help address the identified micronutrient gaps. Most countries in this analysis have a national vitamin A supplementation programme. Some countries have subnational or routine iron supplementation for young children; however, coverage is frequently very low (ASEAN, UNICEF, & WFP, 2022).

Fortification of staple foods and condiments is recognised as an efficient, evidence‐based and cost‐effective intervention to increase micronutrient intake and prevent deficiencies. However, while large‐scale food fortification may be effective at a population level, it may not be ideal for improving micronutrient intake during the complementary feeding period. A systematic review and meta‐analysis of fortification data in low‐ and middle‐income countries found that large‐scale food fortification had a higher impact on the micronutrient status of adults than of young children. Young children may reap fewer benefits from large‐scale fortification in part due to their relatively low consumption of fortified staple foods (small portion sizes) and comparatively high nutrient needs. Additional strategies may be required to ensure adequate micronutrient intake in children aged 6–23 months, such as fortification of complementary foods (Keats et al., 2019).

Point‐of‐use fortification using MNPs can contribute to improved micronutrient intake (Siekmans et al., 2017) and is recommended by the World Health Organization (WHO) to reduce the risk of iron deficiency and anaemia in children aged 6–23 months (WHO, 2016). The use of MNPs should be incorporated into broader infant and young child feeding programming where warranted (Siekmans et al., 2017).

Packaged complementary foods promoted as suitable for children aged 6–23 months—also known as commercially produced complementary foods (CPCF)—if well regulated, could provide micronutrients in a form that is already familiar to caregivers. Fortified CPCF dry or instant cereals, for example, are similar to traditional rice or cereal porridges fed to young children across SEA. The nutrient composition and fortification of CPCF infant cereals available in SEA were recently assessed, with results summarised in a separate paper in this supplement. Approximately two‐thirds of the CPCF infant cereals were fortified; however, the presence of fortification varied by micronutrient: Approximately 60% were fortified with iron, 57% were fortified with calcium, 53% were fortified with zinc, 53% were fortified with vitamin A and 48% were fortified with vitamin D (Bassetti et al., 2023).

Nutrition programmes and policies should consider a multitude of approaches to equitably improve the diets of children aged 6–23 months. The strategies noted here can be implemented concurrently and in a complementary fashion. Efforts to protect, promote and support breastfeeding are also essential to improve diets during the complementary feeding period. Breastfeeding practices remain suboptimal in several SEA countries, with rates of both exclusive and continued breastfeeding <50% in Malaysia, Viet Nam, Lao PDR and Thailand (ASEAN, UNICEF, & WFP, 2022). Social and behaviour change strategies and interventions are also critical to support efforts to reduce micronutrient gaps, including encouraging the consumption of micronutrient‐dense foods.

Despite its utility in synthesising evidence and identifying micronutrient gaps, CONGA has methodological limitations. The literature search conducted for this analysis was not systematic and thus may have missed available evidence sources. Much of the CONGA methodology is standardised to account for disparate evidence sources that likely vary in quality and relevance. However, there is purposeful flexibility built into CONGA to allow researchers to override quantitative or criteria‐based ratings based on a qualitative assessment of the totality of evidence compiled (White et al., 2021). Further, CONGA can only consider evidence currently available. A lack of evidence (either any availability or robustness of what is available) relevant to intake, availability and deficiency for some or all micronutrients of interest severely limits the utility of this tool. The scarcity of data on the prevalence and burden of micronutrient deficiencies—even for iron, vitamin A and zinc—was identified as a limitation of a recent global analysis on micronutrient deficiencies in young children. The population‐based biomarker data that is available is often limited to a minimal selection of micronutrients and/or population groups and is infrequently updated in most countries (Brown et al., 2021; Stevens et al., 2022) in part due to high costs and logistical constraints as well as limited awareness among policymakers of the importance of micronutrient intake and status for health and development and the utility of deficiency and intake data for programme and policy planning. Advocacy is needed with policymakers and donors to highlight the scarcity of relevant data and how this critical gap is impacting planning for nutrition programmes and policies (Brown et al., 2021). The lack of global guidance on thresholds to determine the public health significance of deficiencies in vitamin D, calcium and other micronutrients is also a barrier to the interpretation of the burden of particular micronutrient gaps. As a result, while data on the prevalence of calcium and thiamine deficiency was available in some countries, these indicators were only considered qualitatively in this analysis. Finally, CONGA does not consider breastmilk intake—an important source of nutrients during the complementary feeding period—when assessing nutrient gaps.

## 5 CONCLUSION

Gaps were identified in vitamin D, zinc and iron and a potential gap was identified in calcium in the diets of children aged 6–23 months in SEA. Multiple strategies are required to address these gaps. New data collection and evidence generation on micronutrient availability, intake and deficiency should be prioritised in SEA, particularly for the micronutrients with moderate or high nutrient burden ratings but low evidence certainty (i.e., calcium). Micronutrients with low certainty of evidence across all countries should also be considered during new data collection efforts, including folate, vitamin B_12_, thiamine, niacin, vitamin C and vitamin B_6_. Efforts should be made to consider vitamin D in further evidence generation and analyses on the complementary feeding period.

## AUTHOR CONTRIBUTIONS

Jessica M. White, Elizabeth Drummond, Vasundhara Bijalwan and Anusara Singhkumarwong performed the research. Arvind Betigeri and Jessica Blankenship provided technical guidance and review. Jessica M. White, Elizabeth Drummond and Jessica Blankenship wrote the paper.

## CONFLICT OF INTEREST STATEMENT

The authors declare no conflict of interest.

## Supporting information

Supplementary Table 4: CONGA results by country.

Supporting information.

## Data Availability

The data that supports the findings of this study are available in the Supporting Information material of this article.
