# Supplementary material for: Micronutrient gaps during the complementary feeding period in seven countries in Southeast Asia: A Comprehensive Nutrient Gap Assessment
Source: Matern Child Nutr. 2023 Dec 13;19(Suppl 2):e13577. doi: 10.1111/mcn.13577 (PMC10719053; doi:10.1111/mcn.13577)
Supplement: Supplementary file 2 — Supporting information. [file MCN-19-e13577-s002.docx]

**Supplementary Material**

**Supplementary Table 1: Suggested prevalence/mean ranges for implied nutrient gap burden scores ^a^**

| **Indicator** | **Age** | **Implied nutrient burden rating ^b^** | | | |
| --- | --- | --- | --- | --- | --- |
|  |  | **Negligible** | **Low** | **Moderate** | **High** |
| **Biomarkers** |  |  |  |  |  |
| *Iron* ^c^ |  |  |  |  |  |
| Serum ferritin (< 12 mg/l) ^d^ | < 5 y ^e^ | < 3% | 3–9% | 10–19% | ≥ 20% |
| *Vitamin A* |  |  |  |  |  |
| Serum retinol (< 0.7 mmol/l) ^f^ | 6–71 m | < 3% | 3–9% | 10–19% | ≥ 20% |
| *Zinc* |  |  |  |  |  |
| Serum zinc (< 9.9 mmol/l) ^g^ | < 10 y | < 3% | 3–9% | 10–19% | ≥ 20% |
| *Folate* |  |  |  |  |  |
| Serum folate (< 10 nmol/l) ^h^ | All | < 3% | 3–9% | 10–19% | ≥ 20% |
| *Vitamin B12* |  |  |  |  |  |
| Plasma B12 (< 150 pmol/l) ^h^ | All | < 3% | 3–9% | 10–19% | ≥ 20% |
| *Vitamin D* |  |  |  |  |  |
| Serum 25(OH)D <50 nmol/L ^i^ | All | < 3% | 3–9% | 10–19% | ≥ 20% |
| *Iodine* |  |  |  |  |  |
| Urinary iodine concentration (UIC) (< 100 mg/l) | ≥ 6 y ^j^ | < 25% | 25–49% | 50–74% | ≥ 75% |
| Median UIC (mg/l) ^k^ | ≥ 6 y | ≥ 150 | 100–150 | 50–99 | < 50 |
| Total goiter rate ^k^ | 6–12 y | < 3% | 3–9% | 10–19% | ≥ 20% |
| **Inadequate intake or availability** |  |  |  |  |  |
| Iron, vitamin A, zinc, folate, vitamin B12 | All | < 5% | 5–14% | 15–24% | ≥ 25% |
| Calcium, niacin, thiamine, vitamin B6 | All | < 5% | 5–19% | 20–49% | ≥ 50% |
| Vitamin C | All | < 10% | 10–29% | 30–49% | ≥ 50% |
| **Nutrient-informative food group intake** |  |  |  |  |  |
| Vitamin A-rich foods (past 24 hours) | 6–23 m | >90% | 75-90% | 60-74% | <60% |
| Iron-rich foods (past 24 hours) | 6–23 m | > 70% | 60–70% | 50–59% | < 50% |
| Household iodized salt coverage | All | > 90% | 75–90% | 60–74% | < 60% |
| Household adequately iodized salt coverage (≥ 15 ppm) | *All* | *> 80%* | *65–80%* | *50–64%* | *< 50%* |

*Abbreviation*: UIC, urinary iodine concentration; m, months; y, years.

**^a^** Adapted from Beal et al., 2021.

**^b^** Data reported as % excepted for median UIC

**^C^** If only iron deficiency anaemia is available, similar prevalence ranges as those for iron deficiency were used.

**^d^** Various adjustments for inflammation have differing impacts on iron deficiency prevalence (Engle-Stone, Nankap, Ndjebayi, Erhardt, & Brown, 2013). The World Health Organization (WHO) suggests iron deficiency is not prevalent when < 10% of the population is above the manufacture’s cutoff for soluble transferrin receptor values, even in populations with deficiency ≥ 20% when measured by serum ferritin (World Health Organization, 2017).

**^e^** Cutoff is < 15 mg/l for individuals > 5 years old.

**^f^** Prevalence ranges coincide with WHO recommendations (World Health Organization, 1996). Various adjustments for inflammation have different impacts on vitamin A deficiency prevalence. Unadjusted estimates are typically 11–18 percentage points higher than adjusted estimates in areas with high inflammation (Larson et al., 2017). Retinol binding protein is often used as surrogate for serum retinol, which may not always be appropriate, depending on the population.

**^g^** Morning, nonfasting. Cut off is 8.7 mmol/l in the afternoon, for non-fasting children (Wessells, King, & Brown, 2014).

**^h^** See de Benoist, 2008

^i^ For vitamin D deficiency, there is no global consensus on the population thresholds to determine public health significance, however, recent literature suggests a population with >20% vitamin D deficiency prevalence based on a threshold of serum 25-hydroxyvitamin D (serum 25[OH]D) <30 nmol/L requires public health intervention (Roth et al., 2018). While serum 25[OH]D is the recommended biomarker for assessing vitamin D deficiency, there is currently no global consensus on the threshold to define deficiency (Amrein et al., 2020; Roth et al., 2018). Evidence is clear that serum 25(OH)D <25 or <30 nmol/L is associated with an increased risk of rickets and ostemoalcia, yet the Endocrine Society Task Force on Vitamin D (Holick et al., 2011) and the European Food Safety Authority (EFSA Panel on Dietetic Products & Allergies, 2016) define a threshold of <50 nmol/L for deficiency (which corresponds with the recommended dietary allowance per guidance from the Institute of Medicine) (Amrein et al., 2020; Roth et al., 2018). Further, it was found that the majority of studies assessing vitamin D deficiency in Southeast Asia used <50 nmol/L as the deficiency threshold (Oktaria et al., 2022). Thus, for the purposes of this analysis, the implied nutrient burden prevalence ranges for vitamin D deficiency, using a cutoff of <50 nmol/L, was as follows: prevalence <3% (negligible), 3-9.9% (mild), 10-19.9% (moderate), ≥20% (severe).

**^j^** Excluding pregnant and lactating women

**^k^** To align cutoffs with comparable severity across indicators, we suggested different ranges than those in the WHO recommendations (World Health Organization & Food and Agriculture Organization, 2006). These ranges are not intended to replace WHO guidance on public health severity of iodine deficiency.

**References for Table 1**

Amrein, K., Scherkl, M., Hoffmann, M., Neuwersch-Sommeregger, S., Kostenberger, M., Tmava Berisha, A., . . . Malle, O. (2020). Vitamin D deficiency 2.0: an update on the current status worldwide. *Eur J Clin Nutr, 74*(11), 1498-1513. doi:10.1038/s41430-020-0558-y

Beal, T., White, J. M., Arsenault, J. E., Okronipa, H., Hinnouho, G. M., & Morris, S. S. (2021). Comprehensive Nutrient Gap Assessment (CONGA): A method for identifying the public health significance of nutrient gaps. *Nutr Rev, 79*(Suppl 1), 4-15. doi:10.1093/nutrit/nuaa140

de Benoist, B. (2008). Conclusions of a WHO Technical Consultation on folate and vitamin B12 deficiencies. *Food Nutr Bull, 29*(2 Suppl), S238-244. doi:10.1177/15648265080292S129

EFSA Panel on Dietetic Products, N., & Allergies. (2016). Dietary reference values for vitamin D. *EFSA Journal, 14*(10), e04547. doi:https://doi.org/10.2903/j.efsa.2016.4547

Engle-Stone, R., Nankap, M., Ndjebayi, A. O., Erhardt, J. G., & Brown, K. H. (2013). Plasma ferritin and soluble transferrin receptor concentrations and body iron stores identify similar risk factors for iron deficiency but result in different estimates of the national prevalence of iron deficiency and iron-deficiency anemia among women and children in Cameroon. *J Nutr, 143*(3), 369-377. doi:10.3945/jn.112.167775

Holick, M. F., Binkley, N. C., Bischoff-Ferrari, H. A., Gordon, C. M., Hanley, D. A., Heaney, R. P., . . . Weaver, C. M. (2011). Evaluation, Treatment, and Prevention of Vitamin D Deficiency: an Endocrine Society Clinical Practice Guideline. *The Journal of Clinical Endocrinology & Metabolism, 96*(7), 1911-1930. doi:10.1210/jc.2011-0385

Larson, L. M., Namaste, S. M., Williams, A. M., Engle-Stone, R., Addo, O. Y., Suchdev, P. S., . . . Northrop-Clewes, C. A. (2017). Adjusting retinol-binding protein concentrations for inflammation: Biomarkers Reflecting Inflammation and Nutritional Determinants of Anemia (BRINDA) project. *Am J Clin Nutr, 106*(Suppl 1), 390S-401S. doi:10.3945/ajcn.116.142166

Oktaria, V., Putri, D. A. D., Ihyauddin, Z., Julia, M., Sulistyoningrum, D. C., Koon, P. B., . . . Murni, I. K. (2022). Vitamin D deficiency in South-East Asian children: a systematic review. *Arch Dis Child*. doi:10.1136/archdischild-2021-323765

Roth, D. E., Abrams, S. A., Aloia, J., Bergeron, G., Bourassa, M. W., Brown, K. H., . . . Whiting, S. J. (2018). Global prevalence and disease burden of vitamin D deficiency: a roadmap for action in low- and middle-income countries. *Ann N Y Acad Sci, 1430*(1), 44-79. doi:10.1111/nyas.13968

Wessells, K. R., King, J. C., & Brown, K. H. (2014). Development of a plasma zinc concentration cutoff to identify individuals with severe zinc deficiency based on results from adults undergoing experimental severe dietary zinc restriction and individuals with acrodermatitis enteropathica. *J Nutr, 144*(8), 1204-1210. doi:10.3945/jn.114.191585

World Health Organization. (1996). *Indicators for Assessing Vitamin A Deficiency and Their Application in Monitoring and Evaluating Intervention Programmes*.

World Health Organization. (2017). *Nutritional Anaemias: Tools for Effective Prevention and Control*. Retrieved from

World Health Organization, & Food and Agriculture Organization. (2006). Evaluating the public health significance of micronutrient malnutrition. In Allen L, de Benoist B, Dary O, & H. R (Eds.), *Guidelines on Food Fortification with Micronutrients* (pp. 39–92). Geneva, Switzerland.

**Supplementary Table 2: Certainty-of-evidence rating criteria** **^a^**

| **Low** | **Moderate** | **High** |
| --- | --- | --- |
| ≥1 qualifying data point meeting minimum inclusion criteria for the quantitative nutrient gap burden calculation b | - ≥1 data point with a weight score ^c^ of 51–80 and no disagreements ^d^ with any data point with a weight score > 25 - Or ≥2 data points with a weight score 25–50 and no disagreements with any data point with a weight score > 25 - Or ≥3 data points meeting minimum criteria thresholds, one of which whose weight score is ≥ 15, and no disagreements with any rating | - ≥1 data point with a weight score > 80 and no disagreements with any data point with a weight score > 50 - ≥2 data points with a weight score > 50 and no disagreements with any data point with a weight score > 50 |

^a^ Adapted from Beal et al., 2021.

^b^ Exclusion criteria (1) categorized as ‘other’ evidence, (2) representative of <10% of the national population, (3) collected before 2010, (4) representative of an age and sex group that excluded and is not near to or relevant to children aged 6-23 months, or (5) based on a sample size <50.

^c^ An overall evidence weight score for each data point was calculated by multiplying the weight score of its evidence type metadata by the weight scores of its other four metadata

^d^  A disagreement is a different implied nutrient gap burden score from

**References for Table 2**

Beal, T., White, J. M., Arsenault, J. E., Okronipa, H., Hinnouho, G. M., & Morris, S. S. (2021). Comprehensive Nutrient Gap Assessment (CONGA): A method for identifying the public health significance of nutrient gaps. *Nutr Rev, 79*(Suppl 1), 4-15. doi:10.1093/nutrit/nuaa140

**Supplementary Table 3: Evidence sources used for CONGA, by country**

| **Country** | **Evidence Sources** |
| --- | --- |
| **Cambodia** | Beal, T., Massiot, E., Arsenault, J. E., Smith, M. R., & Hijmans, R. J. (2017). Global trends in dietary micronutrient supplies and estimated prevalence of inadequate intakes. *PLoS One, 12*(4), e0175554. doi:10.1371/journal.pone.0175554  Blaney, S., Menasria, L., Main, B., Chhorvann, C., Vong, L., Chiasson, L., . . . Raminashvili, D. (2019). Determinants of Undernutrition among Young Children Living in Soth Nikum District, Siem Reap, Cambodia. *Nutrients, 11*(3). doi:10.3390/nu11030685  Cambodia National Institute of Statistics, Ministry of Planning,. (2022). *Report of Cambodia Socio-Economic Survey 2021*. Phnom Penh, Cambodia.  Cambodia National Institute of Statistics, Ministry of Planning,, & World Food Programme. (2022). *Food and nutrition security trend analysis report: Cambodia socio-economic survey 2014-2019/20*. Phnom Penh, Cambodia.  Ferguson, E. L., Watson, L., Berger, J., Chea, M., Chittchang, U., Fahmida, U., . . . Winichagoon, P. (2019). Realistic Food-Based Approaches Alone May Not Ensure Dietary Adequacy for Women and Young Children in South-East Asia. *Matern Child Health J, 23*(Suppl 1), 55-66. doi:10.1007/s10995-018-2638-3  Kumssa, D. B., Joy, E. J., Ander, E. L., Watts, M. J., Young, S. D., Walker, S., & Broadley, M. R. (2015). Dietary calcium and zinc deficiency risks are decreasing but remain prevalent. *Sci Rep, 5*, 10974. doi:10.1038/srep10974  Kuong, K., Tor, P., Perignon, M., Fiorentino, M., Chamnan, C., Berger, J., . . . Wieringa, F. T. (2019). Multi-Micronutrient Fortified Rice Improved Serum Zinc and Folate Concentrations of Cambodian School Children. A Double-Blinded Cluster-Randomized Controlled Trial. *Nutrients, 11*(12). doi:10.3390/nu11122843  Laillou, A., Sophonneary, P., Kuong, K., Hong, R., Un, S., Chamnan, C., . . . Wieringa, F. (2016). Low Urinary Iodine Concentration among Mothers and Children in Cambodia. *Nutrients, 8*(4), 172. doi:10.3390/nu8040172  National Institute of Statistics/Cambodia, Directorate General for Health/Cambodia, & ICF International. (2015). *Cambodia Demographic and Health Survey 2014*. Phnom Penh, Cambodia: http://dhsprogram.com/pubs/pdf/FR312/FR312.pdf  Reinbott, A., Jordan, I., Herrmann, J., Kuchenbecker, J., Kevanna, O., & Krawinkel, M. B. (2016). Role of Breastfeeding and Complementary Food on Hemoglobin and Ferritin Levels in a Cambodian Cross-Sectional Sample of Children Aged 3 to 24 Months. *PLoS One, 11*(3), e0150750. doi:10.1371/journal.pone.0150750  Smith, G., Wimalawansa, S. J., Laillou, A., Sophonneary, P., Un, S., Hong, R., . . . Wieringa, F. T. (2016). High Prevalence of Vitamin D Deficiency in Cambodian Women: A Common Deficiency in a Sunny Country. *Nutrients, 8*(5). doi:10.3390/nu8050290  UNICEF Division of Data, Analaysis, Planning and Monitoring. (October 2021). *UNICEF Global Databases on Iodized salt*. New York, New York: UNICEF.  UNICEF Division of Data, Analaysis, Planning and Monitoring. (September 2021). *UNICEF Global database: Vitamin A supplementation coverage 2000-2020 for current priority country list*. New York, New York: UNICEF.  Wessells, K. R., & Brown, K. H. (2012). Estimating the global prevalence of zinc deficiency: results based on zinc availability in national food supplies and the prevalence of stunting. *PLoS One, 7*(11), e50568. doi:10.1371/journal.pone.0050568  Whitfield, K. C., Smith, G., Chamnan, C., Karakochuk, C. D., Sophonneary, P., Kuong, K., . . . Wieringa, F. T. (2017). High prevalence of thiamine (vitamin B1) deficiency in early childhood among a nationally representative sample of Cambodian women of childbearing age and their children. *PLoS Negl Trop Dis, 11*(9), e0005814. doi:10.1371/journal.pntd.0005814  Wieringa, F. T., Dahl, M., Chamnan, C., Poirot, E., Kuong, K., Sophonneary, P., . . . Laillou, A. (2016). The High Prevalence of Anemia in Cambodian Children and Women Cannot Be Satisfactorily Explained by Nutritional Deficiencies or Hemoglobin Disorders. *Nutrients, 8*(6). doi:10.3390/nu8060348  Wieringa, F. T., Sophonneary, P., Whitney, S., Mao, B., Berger, J., Conkle, J., . . . Laillou, A. (2016). Low Prevalence of Iron and Vitamin A Deficiency among Cambodian Women of Reproductive Age. *Nutrients, 8*(4), 197. doi:10.3390/nu8040197 |
| **Indonesia** | Beal, T., Massiot, E., Arsenault, J. E., Smith, M. R., & Hijmans, R. J. (2017). Global trends in dietary micronutrient supplies and estimated prevalence of inadequate intakes. PLoS One, 12(4), e0175554. doi:10.1371/journal.pone.0175554  Fahmida, U., Pramesthi, I. L., Kusuma, S., Wurjandaru, G., & Izwardy, D. (2022). Problem Nutrients and Food-Based Recommendations for Pregnant Women and Under-Five Children in High-Stunting Districts in Indonesia. Curr Dev Nutr, 6(5), nzac028. doi:10.1093/cdn/nzac028  Fahmida, U., Santika, O., Kolopaking, R., & Ferguson, E. (2014). Complementary feeding recommendations based on locally available foods in Indonesia. Food Nutr Bull, 35(4 Suppl), S174-179. doi:10.1177/15648265140354S302  Kartono, D., Atmarita, A., Soekirman, S., & Izwardy, D. (2017). THE SITUATION OF URINARY IODINE CONCENTRATION (UIC) AMONG SCHOOL AGE CHILDREN, WOMEN AT REPRODUCTIVE AGE AND PREGNANT WOMEN IN INDONESIA: THE ANALYSIS OF RISKESDAS 2013. GIZI INDONESIA, 39, 49. doi:10.36457/gizindo.v39i1.207  Kementerian Kemenkes RI. (2014). Laporan Riset Kesehatan Dasar (Riskesdas) 2013. Jakarta, Indonesia.  Kementerian Kemenkes RI. (2019). Laporan Nasional Riskesdas 2018. Jakarta, Indonesia.  Kumssa, D. B., Joy, E. J., Ander, E. L., Watts, M. J., Young, S. D., Walker, S., & Broadley, M. R. (2015). Dietary calcium and zinc deficiency risks are decreasing but remain prevalent. Sci Rep, 5, 10974. doi:10.1038/srep10974  National Population and Family Planning Board (BKKBN), Statistical Indonesia (BPS), Ministry of Health (Kemenkes), & ICF. (2018). Indonesia Demographic and Health Survey 2017. Jakarta, Indonesia: BKKBN, BPS, Kemenkes, and ICF. Available at: http://dhsprogram.com/pubs/pdf/FR342/FR342.pdf  Sandjaja, Jus'at, I., Jahari, A. B., Ifrad, Htet, M. K., Tilden, R. L., . . . Korenromp, E. L. (2015). Vitamin A-fortified cooking oil reduces vitamin A deficiency in infants, young children and women: results from a programme evaluation in Indonesia. Public Health Nutr, 18(14), 2511-2522. doi:10.1017/S136898001400322X  Sandjaja, S., Budiman, B., Harahap, H., Ernawati, F., Soekatri, M., Widodo, Y., . . . Khouw, I. (2013). Food consumption and nutritional and biochemical status of 0.5-12-year-old Indonesian children: the SEANUTS study. Br J Nutr, 110 Suppl 3, S11-20. doi:10.1017/S0007114513002109  Santika, O., Februhartanty, J., & Ariawan, I. (2016). Feeding practices of young children aged 12-23 months in different socio-economic settings: a study from an urban area of Indonesia. Br J Nutr, 116 Suppl 1, S1-7. doi:10.1017/S0007114515003438  Shinta, D., Asmarinah, Adhiyanto, C., Htet, M. K., & Fahmida, U. (2019). The Association of TMPRSS6 Gene Polymorphism and Iron Intake with Iron Status among Under-Two-Year-Old Children in Lombok, Indonesia. Nutrients, 11(4). doi:10.3390/nu11040878  UNICEF Division of Data, Analaysis, Planning and Monitoring,. (October 2021). UNICEF Global Databases on Iodized salt. New York, New York: UNICEF.  Wessells, K. R., & Brown, K. H. (2012). Estimating the global prevalence of zinc deficiency: results based on zinc availability in national food supplies and the prevalence of stunting. PLoS One, 7(11), e50568. doi:10.1371/journal.pone.0050568 |
| **Lao People’s Democratic Republic** | Barffour, M. A., Hinnouho, G. M., Kounnavong, S., Wessells, K. R., Ratsavong, K., Bounheuang, B., . . . Hess, S. Y. (2019). Effects of Daily Zinc, Daily Multiple Micronutrient Powder, or Therapeutic Zinc Supplementation for Diarrhea Prevention on Physical Growth, Anemia, and Micronutrient Status in Rural Laotian Children: A Randomized Controlled Trial. *J Pediatr, 207*, 80-89 e82. doi:10.1016/j.jpeds.2018.11.022  Beal, T., Massiot, E., Arsenault, J. E., Smith, M. R., & Hijmans, R. J. (2017). Global trends in dietary micronutrient supplies and estimated prevalence of inadequate intakes. *PLoS One, 12*(4), e0175554. doi:10.1371/journal.pone.0175554  Ferguson, E. L., Watson, L., Berger, J., Chea, M., Chittchang, U., Fahmida, U., . . . Winichagoon, P. (2019). Realistic Food-Based Approaches Alone May Not Ensure Dietary Adequacy for Women and Young Children in South-East Asia. *Matern Child Health J, 23*(Suppl 1), 55-66. doi:10.1007/s10995-018-2638-3  Kumssa, D. B., Joy, E. J., Ander, E. L., Watts, M. J., Young, S. D., Walker, S., & Broadley, M. R. (2015). Dietary calcium and zinc deficiency risks are decreasing but remain prevalent. *Sci Rep, 5*, 10974. doi:10.1038/srep10974  Hinnouho, G. M., Hampel, D., Shahab-Ferdows, S., Barffour, M. A., McAnena, L., Arnold, C. D., Ryan Wessells, K., Kounnavong, S., Allen, L. H., McNulty, H., & Hess, S. Y. (2022). Daily supplementation of a multiple micronutrient powder improves folate but not thiamine, riboflavin, or vitamin B12 status among young Laotian children: a randomized controlled trial. European journal of nutrition, 61(7), 3423–3435. https://doi.org/10.1007/s00394-022-02890-3  Ministry of Health, & Ministry of Planning and Investment. (2016). *Lao PDR Food and Nutrition Security Survey, 2015: Subnational Household Survey Final Report*.  Ministry of Health/Lao PDR, & Lao Statistics Bureau. (2012). *Lao Social Indicator Survey 2011-12*. Vientiane, Lao PDR. Retrieved from: http://dhsprogram.com/pubs/pdf/FR268/FR268.pdf  Ministry of Health/Lao PDR, Lao Statistics Bureau - LSB, & ICF. (2018). Vientiane, Lao PDR. Retrieved from: http://dhsprogram.com/pubs/pdf/FR356/FR356.pdf  Ratsavong, K., van Elsacker, T., Doungvichit, D., Siengsounthone, L., Kounnavong, S., & Essink, D. (2020). Are dietary intake and nutritional status influenced by gender? The pattern of dietary intake in Lao PDR: a developing country. *Nutr J, 19*(1), 31. doi:10.1186/s12937-020-00545-9  The Iodine Global Network. (2021). *Global scorecard of iodine nutrition in 2020 in the general population based on school-age children (SAC)*. Ottawa, Canada.  UNICEF Division of Data, Analysis, Planning and Monitoring,. (October 2022). *Global UNICEF Global Databases: Infant and Young Child Feeding: Egg and/or flesh food consumption, Minimum dietary diversity, Minimum meal frequency, Minimum acceptable diet.* New York, New York: UNICEF.  UNICEF Division of Data, Analaysis, Planning and Monitoring,. (October 2021). *UNICEF Global Databases on Iodized salt*. New York, New York: UNICEF.  UNICEF Division of Data, Analaysis, Planning and Monitoring,. (September 2021). *UNICEF Global database: Vitamin A supplementation coverage 2000-2020 for current priority country list*. New York, New York: UNICEF.  van der Haar, F., & Gerasimov, G. (February 2015). Laos: renewed commitment to salt iodization. *IDD Newsletter, 43(1)*. Retrieved from <https://ign.org/app/uploads/2023/04/IDD_feb15_mail.pdf>  Wessells, K. R., & Brown, K. H. (2012). Estimating the global prevalence of zinc deficiency: results based on zinc availability in national food supplies and the prevalence of stunting. *PLoS One, 7*(11), e50568. doi:10.1371/journal.pone.0050568 |
| **Malaysia** | ASEAN, UNICEF, & WFP. (2022). *ASEAN Food and Nutrition Security Report, Volume 2*. Retrieved from Jakarta: https://asean.org/book/asean-food-and-nutrition-security-report-2021-volume-1-the-asean-secretariat-jakarta/  Beal, T., Massiot, E., Arsenault, J. E., Smith, M. R., & Hijmans, R. J. (2017). Global trends in dietary micronutrient supplies and estimated prevalence of inadequate intakes. *PLoS One, 12*(4), e0175554. doi:10.1371/journal.pone.0175554  Institute for Public Health (IPH), National Institutes of Health, Ministry of Health Malaysia. (2016). National Health and Morbidity Survey (NHMS) 2016: Maternal and Child Health. Vol. II.  Kuang Kuay, L., Ahmad, N. A., Beng Chin, T., Ying Ying, C., Mahjom, M., Awaluddin, S. M., . . . Aris, T. (2022). A 10-Year Impact Evaluation of the Universal Salt Iodization (USI) Intervention in Sarawak, Malaysia, 2008-2018. *Nutrients, 14*(8). doi:10.3390/nu14081585  Kuay, L. K., Chin, T. B., Ying, C. Y., Hussain, H., Mahmud, N. A., Kassim, M. S. A., . . . Aris, T. (2021). Pregnant Women Are Iodine Deficient While School-Aged Children Demonstrate Adequate Iodine Status in Sarawak, Malaysia. *Food Nutr Bull, 42*(3), 406-413. doi:10.1177/03795721211002079  Kumssa, D. B., Joy, E. J., Ander, E. L., Watts, M. J., Young, S. D., Walker, S., & Broadley, M. R. (2015). Dietary calcium and zinc deficiency risks are decreasing but remain prevalent. *Sci Rep, 5*, 10974. doi:10.1038/srep10974  Poh, B. K., Ng, B. K., Siti Haslinda, M. D., Nik Shanita, S., Wong, J. E., Budin, S. B., . . . Norimah, A. K. (2013). Nutritional status and dietary intakes of children aged 6 months to 12 years: findings of the Nutrition Survey of Malaysian Children (SEANUTS Malaysia). *Br J Nutr, 110 Suppl 3*, S21-35. doi:10.1017/S0007114513002092  Selamat, R., Mohamud, W. N., Zainuddin, A. A., Rahim, N. S., Ghaffar, S. A., & Aris, T. (2010). Iodine deficiency status and iodised salt consumption in Malaysia: findings from a national iodine deficiency disorders survey. *Asia Pac J Clin Nutr, 19*(4), 578-585. Retrieved from https://www.ncbi.nlm.nih.gov/pubmed/21147721  Tan, P. Y., Mohd Johari, S. N., Teng, K. T., Loganathan, R., Lee, S. C., Ngui, R., . . . Lim, Y. A. L. (2023). High prevalence of malnutrition and vitamin A deficiency among schoolchildren of rural areas in Malaysia using a multi-school assessment approach. *Br J Nutr, 129*(3), 454-467. doi:10.1017/S0007114522001398  Wessells, K. R., & Brown, K. H. (2012). Estimating the global prevalence of zinc deficiency: results based on zinc availability in national food supplies and the prevalence of stunting. *PLoS One, 7*(11), e50568. doi:10.1371/journal.pone.0050568 |
| **Philippines** | Angeles-Agdeppa, I., & Tanda, K. V. (2021). Vitamin D Status and Usual Nutrient Intake of Filipino Children Aged 6–12 Years in Selected Areas in the Philippines: A 2018 National Nutrition Survey. *Journal of Nutrition and Metabolism, 2021*, 8515607. doi:10.1155/2021/8515607  Beal, T., Massiot, E., Arsenault, J. E., Smith, M. R., & Hijmans, R. J. (2017). Global trends in dietary micronutrient supplies and estimated prevalence of inadequate intakes. *PLoS One, 12*(4), e0175554. doi:10.1371/journal.pone.0175554  Denney, L., Angeles-Agdeppa, I., Capanzana, M. V., Toledo, M. B., Donohue, J., & Carriquiry, A. (2018). Nutrient Intakes and Food Sources of Filipino Infants, Toddlers and Young Children are Inadequate: Findings from the National Nutrition Survey 2013. *Nutrients, 10*(11). doi:10.3390/nu10111730  Department of Science and Technology - Food and Nutrition Research Institute. (2020). *Philippine Nutrition Facts and Figures: 2018 Expanded National Nutrition Survey (ENNS)*. Manila, Philippines.  Food and Nutrition Research Institute-Department of Science and Technology. (2015a). *2nd NATIONAL NUTRITION SUMMIT: 8th NATIONAL NUTRITION SURVEY*. Manila, Philippines.  Food and Nutrition Research Institute-Department of Science and Technology. (2015b). *Philippine Nutrition Facts and Figures 2013: Biochemical Survey, 2nd Edition*. Manila, Philippines.  Kumssa, D. B., Joy, E. J., Ander, E. L., Watts, M. J., Young, S. D., Walker, S., & Broadley, M. R. (2015). Dietary calcium and zinc deficiency risks are decreasing but remain prevalent. *Sci Rep, 5*, 10974. doi:10.1038/srep10974  Mejos, K. K., Ignacio, M. S., Jayasuriya, R., & Arcot, J. (2021). Use of Linear Programming to Develop Complementary Feeding Recommendations to Improve Nutrient Adequacy and Dietary Diversity Among Breastfed Children in the Rural Philippines. *Food Nutr Bull, 42*(2), 274-288. doi:10.1177/0379572121998125  Ramirez, M. A. R., & Ducay, A. J. (2021). Determinants of normal nutrition among 0-59-month-old Filipino children living in low-income households. *Nutr Health, 27*(4), 423-434. doi:10.1177/0260106021992669  Rohner, F., Woodruff, B. A., Aaron, G. J., Yakes, E. A., Lebanan, M. A., Rayco-Solon, P., & Saniel, O. P. (2013). Infant and young child feeding practices in urban Philippines and their associations with stunting, anemia, and deficiencies of iron and vitamin A. *Food Nutr Bull, 34*(2 Suppl), S17-34. doi:10.1177/15648265130342S104  Wessells, K. R., & Brown, K. H. (2012). Estimating the global prevalence of zinc deficiency: results based on zinc availability innational food supplies and the prevalence of stunting. *PLoS One, 7*(11), e50568. doi:10.1371/journal.pone.0050568 |
| **Thailand** | Beal, T., Massiot, E., Arsenault, J. E., Smith, M. R., & Hijmans, R. J. (2017). Global trends in dietary micronutrient supplies and estimated prevalence of inadequate intakes. *PLoS One, 12*(4), e0175554. doi:10.1371/journal.pone.0175554  Chotivichien, S., Chongchaithet, N., Aksornchu, P., Boonmongkol, N., Duangmusik, P., Knowles, J., & Sinawat, S. (2021). Assessment of the contribution of industrially processed foods to salt and iodine intake in Thailand. *PLoS One, 16*(7), e0253590. doi:10.1371/journal.pone.0253590  Ferguson, E. L., Watson, L., Berger, J., Chea, M., Chittchang, U., Fahmida, U., . . . Winichagoon, P. (2019). Realistic Food-Based Approaches Alone May Not Ensure Dietary Adequacy for Women and Young Children in South-East Asia. *Matern Child Health J, 23*(Suppl 1), 55-66. doi:10.1007/s10995-018-2638-3  National Statistical Office, & United Nations Children’s Fund. (2016). *Thailand Multiple Indicator Cluster Survey 2015- 2016, Final Report*. Bangkok, Thailand.  National Statistical Office of Thailand. (2020). *Thailand Multiple Indicator Cluster Survey 2019, Survey Findings Report*. Bangkok, Thailand.  Reesukumal, K., Manonukul, K., Jirapongsananuruk, O., Krobtrakulchai, W., Hanyongyuth, S., Chatsiricharoenkul, S., & Pratumvinit, B. (2015). Hypovitaminosis D in healthy children in Central Thailand: prevalence and risk factors. *BMC Public Health, 15*, 248. doi:10.1186/s12889-015-1588-6  Rojroongwasinkul, N., Kijboonchoo, K., Wimonpeerapattana, W., Purttiponthanee, S., Yamborisut, U., Boonpraderm, A., . . . Khouw, I. (2013). SEANUTS: the nutritional status and dietary intakes of 0.5-12-year-old Thai children. *Br J Nutr, 110 Suppl 3*, S36-44. doi:10.1017/S0007114513002110  The Iodine Global Network. (2021). *Global scorecard of iodine nutrition in 2020 in the general population based on school-age children (SAC)*. Ottawa, Canada.  UNICEF Division of Data, Analysis, Planning and Monitoring,. (October 2022). *Global UNICEF Global Databases: Infant and Young Child Feeding: Egg and/or flesh food consumption, Minimum dietary diversity, Minimum meal frequency, Minimum acceptable diet.* New York, New York: UNICEF.  Yanola, J., Kongpan, C., & Pornprasert, S. (2014). Prevalence of anemia, iron deficiency, thalassemia and glucose-6-phosphate dehydrogenase deficiency among hill-tribe school children in Omkoi District, Chiang Mai Province, Thailand. *Southeast Asian J Trop Med Public Health, 45*(4), 920-925. Retrieved from <https://www.ncbi.nlm.nih.gov/pubmed/25427361>  2014-15 Thailand National Health Examination Survey (NHES)  (2019). A survey on prevalence and risk for vitamin A deficiency among children under 5 years old in southern border provinces, Thailand. Retrieved from: https://thaidj.org/index.php/JHS/article/view/9196/8350 (Report in Thai language, reviewed with assistance). |
| **Viet Nam** | Beal, T., Massiot, E., Arsenault, J. E., Smith, M. R., & Hijmans, R. J. (2017). Global trends in dietary micronutrient supplies and estimated prevalence of inadequate intakes. PLoS One, 12(4), e0175554. doi:10.1371/journal.pone.0175554  Ferguson, E. L., Watson, L., Berger, J., Chea, M., Chittchang, U., Fahmida, U., . . . Winichagoon, P. (2019). Realistic Food-Based Approaches Alone May Not Ensure Dietary Adequacy for Women and Young Children in South-East Asia. Matern Child Health J, 23(Suppl 1), 55-66. doi:10.1007/s10995-018-2638-3  General Statistical Office. (2011). Viet Nam Multiple Indicator Cluster Survey 2011, Final Report. Hanoi, Viet Nam.  Kumssa, D. B., Joy, E. J., Ander, E. L., Watts, M. J., Young, S. D., Walker, S., & Broadley, M. R. (2015). Dietary calcium and zinc deficiency risks are decreasing but remain prevalent. Sci Rep, 5, 10974. doi:10.1038/srep10974  Laillou, A., Pham, T. V., Tran, N. T., Le, H. T., Wieringa, F., Rohner, F., . . . Berger, J. (2012). Micronutrient deficits are still public health issues among women and young children in Vietnam. PLoS One, 7(4), e34906. doi:10.1371/journal.pone.0034906  Laillou, A., Wieringa, F., Tran, T. N., Van, P. T., Le, B. M., Fortin, S., . . . Berger, J. (2013). Hypovitaminosis D and mild hypocalcaemia are highly prevalent among young Vietnamese children and women and related to low dietary intake. PLoS One, 8(5), e63979. doi:10.1371/journal.pone.0063979  Le Nguyen, B. K., Le Thi, H., Nguyen Do, V. A., Tran Thuy, N., Nguyen Huu, C., Thanh Do, T., . . . Khouw, I. (2013). Double burden of undernutrition and overnutrition in Vietnam in 2011: results of the SEANUTS study in 0.5-11-year-old children. Br J Nutr, 110 Suppl 3, S45-56. doi:10.1017/S0007114513002080  National Institute of Nutrition Viet Nam. (2015). Micronutrient deficiencies among children and women in Vietnam.  National Institute of Nutrition Viet Nam. (2020). Main findings of general nutrition survey 2019-2020. Ha Noi, Viet Nam.  UNICEF. (2021). WHO and UNICEF call on Vietnamese authorities to enforce food fortification regulations [Press release]. Retrieved from https://www.unicef.org/vietnam/press-releases/who-and-unicef-call-vietnamese-authorities-enforce-food-fortification-regulations  UNICEF Division of Data, Analysis, Planning and Monitoring,. (October 2021). UNICEF Global Databases on Iodized salt. New York, New York: UNICEF.  Wessells, K. R., & Brown, K. H. (2012). Estimating the global prevalence of zinc deficiency: results based on zinc availability in national food supplies and the prevalence of stunting. PLoS One, 7(11), e50568. doi:10.1371/journal.pone.0050568  Wirth, J. P., Petry, N., Tanumihardjo, S. A., Rogers, L. M., McLean, E., Greig, A., . . . Rohner, F. (2017). Vitamin A Supplementation Programs and Country-Level Evidence of Vitamin A Deficiency. Nutrients, 9(3). doi:10.3390/nu9030190 |
